# Elementary Vectors and Conformal Sums in Polyhedral Geometry and their Relevance for Metabolic Pathway Analysis

**DOI:** 10.3389/fgene.2016.00090

**Published:** 2016-05-24

**Authors:** Stefan Müller, Georg Regensburger

**Affiliations:** Radon Institute for Computational and Applied Mathematics, Austrian Academy of SciencesLinz, Austria

**Keywords:** Minkowski's theorem, Carathéodory's theorem, s-cone, polyhedral cone, polyhedron, conformal generators

## Abstract

A fundamental result in metabolic pathway analysis states that every flux mode can be decomposed into a sum of elementary modes. However, only a decomposition without cancelations is biochemically meaningful, since a reversible reaction cannot have different directions in the contributing elementary modes. This essential requirement has been largely overlooked by the metabolic pathway community. Indeed, every flux mode can be decomposed into elementary modes without cancelations. The result is an immediate consequence of a theorem by Rockafellar which states that every element of a linear subspace is a conformal sum (a sum without cancelations) of elementary vectors (support-minimal vectors). In this work, we extend the theorem, first to “subspace cones” and then to general polyhedral cones and polyhedra. Thereby, we refine Minkowski's and Carathéodory's theorems, two fundamental results in polyhedral geometry. We note that, in general, elementary vectors need not be support-minimal; in fact, they are conformally non-decomposable and form a unique minimal set of conformal generators. Our treatment is mathematically rigorous, but suitable for systems biologists, since we give self-contained proofs for our results and use concepts motivated by metabolic pathway analysis. In particular, we study cones defined by linear subspaces and nonnegativity conditions — like the flux cone — and use them to analyze general polyhedral cones and polyhedra. Finally, we review applications of elementary vectors and conformal sums in metabolic pathway analysis.

## 1. Introduction

Cellular metabolism is the set of biochemical reactions which transform nutrients from the environment into all the biomolecules a living cell consists of. Most metabolic reactions are catalyzed by enzymes, the expression and activity of which is controlled by gene and allosteric regulation, respectively.

A metabolic network together with enzymatic reaction rates gives rise to a nonlinear dynamical system for the metabolite concentrations. However, for genome-scale networks, quantitative knowledge of the underlying kinetics is not available, and a mathematical analysis is not practicable. Instead, one considers only stoichiometric information and studies the system of linear equalities and inequalities for the fluxes (net reaction rates), arising from the pseudo steady-state assumption and irreversibility constraints.

A metabolic network is given by *n* internal metabolites, *r* reactions, and the corresponding stoichiometric matrix *N* ∈ ℝ^*n*×*r*^, which contains the net stoichiometric coefficients of each metabolite in each reaction. The set of irreversible reactions is given by I ⊆ {1, …, *r*}. One is interested in the *flux cone*

$$C=\left\{f\in {\mathbb{R}}^{r}|Nf=0\text{and}f_{i}\geq 0\text{for}i\in I\right\},$$

which is a polyhedral cone defined by the null-space of the stoichiometric matrix and nonnegativity conditions. Its elements are called *flux modes*.

As a running example, we consider a small network, taken from Schuster et al. (2002), the corresponding stoichiometric matrix, and the resulting flux cone:

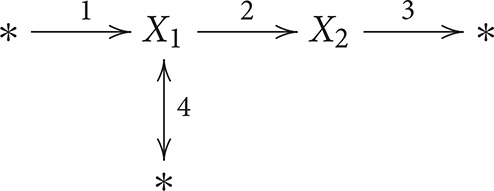



$$N=\left(\begin{array}{cccc} 1 & -1 & 0 & -1\\ 0 & 1 & -1 & 0 \end{array}\right),$$

$$\begin{array}{l} C=\left\{f\in {\mathbb{R}}^{4}|Nf=0\text{ and }f_{1},f_{2},f_{3}\geq 0\right\}. \end{array}$$

The network consists of two internal metabolites *X*_1_, *X*_2_ and four chemical reactions. Reaction 1 imports *X*_1_ from the environment (indicated by the symbol ^*^) which yields the first column (1, 0)^*T*^ of the stoichiometric matrix *N*. Reaction 2 transforms *X*_1_ into *X*_2_ which gives the column (−1, 1)^*T*^, and reaction 3 exports *X*_2_ which gives (0, −1)^*T*^. The first three reactions are assumed to be irreversible which yields the nonnegativity constraints *f*_1_, *f*_2_, *f*_3_ ≥ 0 in the definition of the flux cone *C*. Finally, reaction 4 is reversible and exports/imports *X*_1_.

Metabolic pathway analysis aims to identify biochemically/biologically/biotechnologically meaningful routes in a network, in particular, the smallest routes. Several definitions for minimal metabolic pathways have been given in the literature, with *elementary modes* (EMs) being the fundamental concept both biologically and mathematically Klamt and Stelling (2003); Llaneras and Picó (2010). Formally, EMs are defined as support-minimal (or, equivalently, support-wise non-decomposable) flux modes Schuster and Hilgetag (1994); Schuster et al. (2002). Clearly, a positive multiple of an EM is also an EM since it fulfills the steady-state condition and the irreversibility constraints.

In the example, the EMs are given by *e*^1^ = (1, 0, 0, 1)^*T*^, *e*^2^ = (0, 1, 1, −1)^*T*^, *e*^3^ = (1, 1, 1, 0)^*T*^, and their positive multiples. It is easy to check that *e*^1^, *e*^2^, and *e*^3^ are flux modes (elements of the flux cone) and support-minimal. Note that *e*^3^ = *e*^1^ + *e*^2^.

A fundamental result in metabolic pathway analysis states that every flux mode can be decomposed into a sum of EMs Schuster et al. (2002). However, only a decomposition without cancelations is biochemically meaningful, since a reversible reaction cannot have different directions in the contributing EMs. This essential requirement has been largely overlooked by the metabolic pathway community. Indeed, as we will show in this work, every flux mode can be decomposed into EMs *without cancelations*, that is,

(0) if a component of the flux mode is zero, then this component is zero in the contributing EMs,
(+) if a component of the flux mode is positive, then this component is positive or zero in the contributing EMs,
(−) if a component of the flux mode is negative, then this component is negative or zero in the contributing EMs.

In mathematical terms, every nonzero element of a “subspace cone” (defined by a linear subspace and nonnegativity conditions) is a conformal sum of elementary vectors, cf. Theorem 3. The result is stated in Urbanczik and Wagner (2005) and Urbanczik (2007); part (0) has been shown in Schuster et al. (2002) and guarantees a decomposition without cancelations in a weaker sense Llaneras and Picó (2010); Zanghellini et al. (2013).

In the example, the flux mode *f* = (2, 1, 1, 1)^*T*^ can be decomposed into EMs in two ways:

$$\begin{array}{l} f=\left(\begin{array}{c} 2\\ 1\\ 1\\ 1 \end{array}\right)=2e^{1}+e^{2}=\left(\begin{array}{c} 2\\ 0\\ 0\\ 2 \end{array}\right)+\left(\begin{array}{c} 0\\ 1\\ 1\\ -1 \end{array}\right)\\ \;=e^{1}+e^{3}=\left(\begin{array}{c} 1\\ 0\\ 0\\ 1 \end{array}\right)+\left(\begin{array}{c} 1\\ 1\\ 1\\ 0 \end{array}\right). \end{array}$$

The first sum involves a cancelation in the last component of the flux. The last reaction is reversible, however, it cannot have a net rate in different directions at the same time. Hence, only the second sum is biochemically meaningful. As stated above, a decomposition without cancelations is always possible.

In convex analysis, elementary vectors of a linear subspace were introduced as support-minimal vectors by Rockafellar in 1969. He proves that every vector is a conformal sum (originally called harmonious superposition) of elementary vectors (Rockafellar, 1969, Theorem 1). For proofs and generalizations in the settings of polyhedral geometry and oriented matroids (see Ziegler, 1995, Lemma 6.7) and (Bachem and Kern, 1992, Theorem 5.36). Rockafellar points out that this result is easily shown to be equivalent to Minkowski's theorem Minkowski (1896) for pointed polyhedral cones, stating that every nonzero vector is a nonnegative linear combination of extreme vectors. Moreover, the result immediately implies Carathéodory's theorem Carathéodory (1911), stating that the number of extreme vectors in such a nonnegative linear combination need not exceed the dimension of the cone. In fact, Rockafellar writes: “This is even a convenient route for attaining various important facts about polyhedral convex cones, since the direct proof […] for Theorem 1 is so elementary.”

In metabolic pathway analysis, decompositions without cancelations were introduced by Urbanczik and Wagner (2005). The corresponding elementary vectors are defined by intersecting a polyhedral cone with all closed orthants of maximal dimension. By applying Minkowski's theorem for pointed polyhedral cones, every vector is a sum of extreme vectors without cancelations. Urbanczik further extended this approach to polyhedra arising from flux cones and inhomogeneous constraints Urbanczik (2007).

In polyhedral geometry, it seems that conformal decompositions of general cones and polyhedra have not yet been studied. In this work, following Rockafellar, we first extend his result to cones defined by linear subspaces and nonnegativity conditions (Theorem 3). For subspace cones, support-minimality is equivalent to conformal non-decomposability. As it turns out, for general polyhedral cones, elementary vectors have to be defined as conformally non-decomposable vectors. However, these are in one-to-one correspondence with elementary vectors of a higher-dimensional subspace cone, and, by our result for subspace cones, we obtain a conformal refinement of Minkowski's and Carathéodory's theorems for polyhedral cones (Theorem 8). In particular, there is an upper bound on the number of elementary vectors needed in a conformal decomposition of a vector. Finally, by taking into account vertices and conformal convex combinations, we further extend our result to polyhedra (Theorem 13). We note that elementary vectors do not form a minimal generating set (of an s-cone, a general polyhedral cone, or a polyhedron). However, they form a unique minimal set of *conformal* generators (Proposition 17).

## 2. Definitions

We denote the nonnegative real numbers by ℝ_≥_. For *x* ∈ ℝ^*n*^, we write *x* ≥ 0 if $x\in {\mathbb{R}}_{\geq }^{n}$. Further, we denote the *support* of a vector *x* ∈ ℝ^*n*^ by supp(*x*) = {*i* ∣ *x*_*i*_ ≠ 0}.

### 2.1. Sign vectors

For *x* ∈ ℝ^*n*^, we define the *sign vector* sign(*x*) ∈ {−, 0, +}^*n*^ by applying the sign function component-wise, that is, sign(*x*)_*i*_ = sign(*x*_*i*_) for *i* = 1, …, *n*. The relations 0 < − and 0 < + induce a partial order on {−, 0, +}^*n*^: for *X, Y* ∈ {−, 0, +}^*n*^, we write *X* ≤ *Y* if the inequality holds component-wise. For *x, y* ∈ ℝ^*n*^, we say that *x conforms to y*, if sign(*x*) ≤ sign(*y*). For example, let *x* = (−1, 0, 2)^*T*^ and *y* = (−2, −1, 1). Then,

$$\text{sign ​}\left(\begin{array}{c} -1\\ 0\\ 2 \end{array}\right)=\left(\begin{array}{c} -\\ 0\\ + \end{array}\right)\leq \left(\begin{array}{c} -\\ -\\ + \end{array}\right)=\text{sign ​}\left(\begin{array}{c} -2\\ -1\\ 1 \end{array}\right),$$

that is, sign(*x*) ≤ sign(*y*), and *x* conforms to *y*. Let *X* ∈ {−, 0, +}^*n*^. The corresponding closed orthant *O* ⊂ ℝ^*n*^ is defined as *O* = {*x* ∣ sign(*x*) ≤ *X*}.

### 2.2. Convex cones

A nonempty subset *C* of a vector space is a *convex cone*, if

x,y∈C and μ,ν>0 imply μx+νy∈C,

or, equivalently, if

λC=C for all λ>0 and C+C=C.

A convex cone *C* is called *pointed* if *C*∩−*C* = {0}. It is *polyhedral* if

$$C=\left\{x|Ax\geq 0\right\}\text{ for some }A\in {\mathbb{R}}^{m\times r},$$

that is, if it is defined by finitely many homogeneous inequalities. Hence, a polyhedral cone is pointed if and only if ker(*A*) = {0}.

### 2.3. Special vectors

We recall the definitions of support-minimal vectors and extreme vectors, which play an important role in both polyhedral geometry and metabolic pathway analysis. We also introduce support-wise non-decomposable vectors, which serve as elementary modes for flux cones (in the original definition), and conformally non-decomposable vectors, which serve as elementary vectors for general polyhedral cones (see Subsection 3.2).

Let *C* be a convex cone. A nonzero vector *x* ∈ *C* is called

- *support-minimal*, if
  (SM) $$\begin{array}{ll} \text{for all nonzero }x\prime \in C,\; & \;\\ \text{supp}\left(x\prime \right)\subseteq \text{supp}\left(x\right)\text{ implies supp}\left(x\prime \right)=\text{supp}\left(x\right), \end{array}$$
- *support-wise non-decomposable*, if
  (swND) $$\begin{array}{l} \text{for all nonzero }x^{1},x^{2}\in C\text{ with supp}\left(x^{1}\right),\text{supp}\left(x^{2}\right)\subseteq \text{supp}\left(x\right),\\ x=x^{1}+x^{2}\text{ implies supp}\left(x^{1}\right)=\text{supp}\left(x^{2}\right), \end{array}$$
- *conformally non-decomposable*, if
  (cND) $$\begin{array}{l} \text{for all nonzero }x^{1},x^{2}\in C\text{ with sign}\left(x^{1}\right),\text{sign}\left(x^{2}\right)\leq \text{sign}\left(x\right),\\ x=x^{1}+x^{2}\text{ implies }x^{1}=\lambda x^{2}\text{ with }\lambda >0, \end{array}$$
- and *extreme*, if
  (EX) $$\begin{array}{l} \text{for all nonzero }x^{1},x^{2}\in C,\\ x=x^{1}+x^{2}\text{ implies }x^{1}=\lambda x^{2}\text{ with }\lambda >0. \end{array}$$

From the definitions, we have the implications

SM⇒swND⇐EX⇒cND.

If *x* ∈ *C* is extreme, then {λ*x* ∣ λ > 0} is called an extreme ray of *C*. In fact, *C* has an extreme ray if and only if *C* is pointed. If *C* is contained in a closed orthant (and hence pointed), we have the equivalence cND ⇔ EX.

## 3. Mathematical results

We start by extending a result on conformal decompositions into elementary vectors from linear subspaces to special cases of polyhedral cones, including flux cones in metabolic pathway analysis.

### 3.1. Linear subspaces and s-cones

We consider linear subspaces with optional nonnegativity constraints as special cases of polyhedral cones. Let *S* ⊆ ℝ^*r*^ be a linear subspace and 0 ≤ *d* ≤ *r*. We define the resulting s-cone (subspace cone, special cone) as

$$C\left(S,d\right)=\left\{\left(\begin{array}{c} x\\ y \end{array}\right)\in {\mathbb{R}}^{\left(r-d\right)+d}|\left(\begin{array}{c} x\\ y \end{array}\right)\in S,y\geq 0\right\}.$$

Clearly, *C*(*S*, 0) = *S* and $C\left(S,r\right)=S\cap {\mathbb{R}}_{\geq }^{r}$.

**Definition 1**. *Let C(S, d) be an s-cone. A vector e ∈ C(S, d) is called* elementary *if it is support-minimal*.

For linear subspaces, the definition of elementary vectors (EVs) as SM vectors was given in Rockafellar (1969). For flux cones, where *S* = ker(*N*), the definition of elementary modes (EMs) as SM vectors was given in Schuster et al. (2002). Interestingly, the choice of the same adjective for the closely related concepts of elementary vectors and elementary modes was coincidental Schuster (2015).

In the proofs of Theorem 3 and Propositions 4 and 5, we use the following argument.

**Lemma 2**. *Let C(S, d) be an s-cone and x, x′ ∈ C(S, d) be nonzero vectors which are not proportional. If* supp(*x*′) ⊆ supp(*x*), *then there exists a nonzero vector*

x″=x-λx′∈C(S,d)  with λ∈ℝ

such that

sign(x″)≤sign(x)  and  supp(x″)⊂supp(x).

*If* sign(*x*′) ≤ sign(*x*), *then* λ > 0 *in x*″.

*Proof*. Clearly, *x*″ = *x* − λ*x*′ is nonzero for all λ ∈ ℝ. There exists a largest λ > 0 (in case sign(−*x*′) ≤ sign(*x*) a smallest λ < 0) such that sign(*x*″) ≤ sign(*x*). For this λ, *x*″ ∈ *C*(*S, d*) and supp(*x*″) ⊂ supp(*x*).                         □

For linear subspaces, the following fundamental result was proved in Rockafellar (1969 Theorem 1). We extend it to s-cones.

**Theorem 3**. *Let C(S, d) be an s-cone. Every nonzero vector x ∈ C(S, d) is a conformal sum of EVs. That is, there exists a finite set E ⊆ C(S, d) of EVs such that*

$$x=\underset{e\in E}{\sum }e\;\mathrm{with}\text{ sign}\left(e\right)\leq \text{sign}\left(x\right).$$

*The set E can be chosen such that its elements are linearly independent, in particular, they can be ordered such that every e ∈ E has a component which is nonzero in e, but zero in its predecessors (in the ordered set). Then, |E| ≤* dim(*S*) *and |E| ≤ |*supp(*x*)|.

*Proof*. We proceed by induction on the cardinality of supp(*x*).

Either, *x* is SM (and *E* = {*x*}) or there exists a nonzero vector *x*′ ∈ *C*(*S, d*) with supp(*x*′) ⊂ supp(*x*), but not necessarily with sign(*x*′) ≤ sign(*x*). However, by Lemma 2, there exists a nonzero vector *x*″ ∈ *C*(*S, d*) with sign(*x*″) ≤ sign(*x*) and supp(*x*″) ⊂ supp(*x*). By the induction hypothesis, there exists a SM vector e^*^ with sign(e^*^) ≤ sign(*x*″) and hence sign(e^*^) ≤ sign(*x*). By Lemma 2 again, there exists a nonzero vector

$$x^{*}=x-\lambda e^{*}\in C\left(S,d\right)\text{ with }\lambda >0$$

such that sign(x^*^) ≤ sign(*x*) and supp(x^*^) ⊂ supp(*x*). By the induction hypothesis, there exists a finite set E^*^ of SM vectors such that

$$x^{*}=\underset{e\in E^{*}}{\sum }e\text{ with sign}\left(e\right)\leq \text{sign}\left(x^{*}\right)$$

and hence sign(*e*) ≤ sign(*x*). We have constructed a finite set *E* = E^*^ ∪ {λe^*^} of SM vectors such that

$$x=x^{*}+\lambda e^{*}=\underset{e\in E^{*}}{\sum }e+\lambda e^{*}=\underset{e\in E}{\sum }e\text{ with sign}\left(e\right)\leq \text{sign}\left(x\right).$$

By the induction hypothesis, the set E^*^ can be chosen such that its elements are linearly independent and ordered such that every *e* ∈ E^*^ has a component which is nonzero in *e*, but zero in all its predecessors. By construction, λe^*^ has a component which is nonzero, but zero in x^*^ and hence in all *e* ∈ E^*^. Obviously, the elements of *E* = E^*^ ∪ {λe^*^} are linearly independent and can be ordered accordingly.                         □

The statement about the support of the EVs was too strong in Rockafellar (1969, Theorem 1). It was claimed that every EV has a nonzero component which is zero in all other EVs.^*^

Theorem 3 is a conformal refinement of Minkowski's and Carathéodory's theorems for s-cones. In fact, it remains to show that there are finitely many EVs.

**Proposition 4**. *Let C(S, d) be an s-cone. If two SM vectors x, x′ ∈ C(S, d) have the same sign vector, sign(x) = sign(x′), then x = λx′ with* λ > 0. *As a consequence, there are finitely many SM vectors up to positive scalar multiples*.

*Proof*. Assume there are two SM vectors with the same sign vector which are not proportional. Then, by Lemma 2, there exists a vector with smaller support.                         □

We conclude by showing that, for s-cones, EVs can be equivalently defined as SM, swND, or cND vectors.

**Proposition 5**. *For an s-cone, support-minimality, support-wise non-decomposability, and conformal non-decomposability are equivalent. That is*,

s-cone: SM⇔swND⇔cND.

*Proof*. SM ⇒ swND: By definition.

swND ⇒ cND: Let *C*(*S, d*) be an s-cone and assume that *x* ∈ *C*(*S, d*) is conformally decomposable, that is, *x* = *x*^1^ + *x*^2^ with nonzero *x*^1^, *x*^2^ ∈ *C*(*S, d*), sign(*x*^1^), sign(*x*^2^) ≤ sign(*x*), and *x*^1^, *x*^2^ being not proportional. By Lemma 2, there exists a nonzero *x*′ = *x* − λ*x*^1^ ∈ *C*(*S, d*) such that supp(*x*′) ⊂ supp(*x*). Hence supp(*x*′) ≠ supp(*x*^1^), and *x* = *x*′ + λ*x*^1^ is support-wise decomposable.

cND ⇒ SM: Let *C*(*S, d*) be an s-cone and assume that *x* ∈ *C*(*S, d*) is not SM, that is, there exists a nonzero *x* ′ ∈ *C*(*S, d*) with supp(*x* ′) ⊂ supp(*x*). Then, there exists a largest λ > 0 such that $x^{1}=\frac{1}{2}x+\lambda x^{\prime }$ and $x^{2}=\frac{1}{2}x-\lambda x^{\prime }$ fulfill sign(*x*^1^), sign(*x*^2^) ≤ sign(*x*). For this λ, either supp(*x*^1^) ⊂ supp(*x*) or supp(*x*^2^) ⊂ supp(*x*); in any case, *x*^1^, *x*^2^ ∈ *C*(*S, d*) and supp(*x*^1^) ≠ supp(*x*^2^). Hence, *x* = *x*^1^ + *x*^2^ is conformally decomposable.                         □

If an s-cone is contained in a closed orthant, then further cND ⇔ EX, and all definitions of special vectors are equivalent.

### 3.2. General polyhedral cones

Let *C* be a polyhedral cone, that is,

$$C=\left\{x\in {\mathbb{R}}^{r}|Ax\geq 0\right\}\text{ for some }A\in {\mathbb{R}}^{m\times r}.$$

For s-cones, we defined elementary vectors (EVs) via support-minimality which, in this case, turned out to be equivalent to conformal non-decomposability. For general polyhedral cones, only the latter concept allows to extend Theorem 3.

**Definition 6**. *Let C be a polyhedral cone. A vector e ∈ C is called* elementary *if it is conformally non-decomposable*.

In order to apply Theorem 3, we define an s-cone related to a polyhedral cone *C*. We introduce the subspace

$$\tilde{S}=\left\{\left(\begin{array}{c} x^{}\\ Ax^{} \end{array}\right)\in {\mathbb{R}}^{r+m}|x\in \text{span}\left(C\right)\right\}$$

with $\text{dim}\left(\tilde{S}\right)=\text{dim}\left(C\right)$ and the s-cone

$$\begin{array}{l} \tilde{C}=C(\tilde{S},m)\\ \;=\left\{\left(\begin{array}{c} x\\ Ax \end{array}\right)\in {\mathbb{R}}^{r+m}|x\in \text{span}(C)\text{ and }Ax\geq 0\right\}\\ \;=\{\left(\begin{array}{c} x\\ Ax \end{array}\right)\in {\mathbb{R}}^{r+m}|x\in C\}. \end{array}$$

Hence,

$$x\in C\;\Leftrightarrow \;\left(\begin{array}{c} x^{}\\ Ax^{} \end{array}\right)\in \tilde{C}.$$

Moreover, the cND vectors of *C* and $\tilde{C}$ are in one-to-one correspondence.

**Lemma 7**. *Let C* = {*x* ∣ *Ax* ≥ 0} *be a polyhedral cone and* $\tilde{C}=\left\{\left(\begin{array}{c} x^{}\\ Ax^{} \end{array}\right)|Ax\geq 0\right\}$ *the related s-cone. Then*,

$$x\in C\text{ is cND }\Leftrightarrow \;\left(\begin{array}{c} x^{}\\ Ax^{} \end{array}\right)\in \tilde{C}\text{ is cND}.$$

*Proof*. First, we show the equivalence of the premises in the definitions of conformal non-decomposability for *C* and $\tilde{C}$. Indeed,

$$\begin{array}{l} x=x^{1}+x^{2}\text{ with }x^{1},x^{2}\in C\\ \Leftrightarrow \\ \left(\begin{array}{c} x^{}\\ Ax^{} \end{array}\right)=\left(\begin{array}{c} x^{1}\\ Ax^{1} \end{array}\right)+\left(\begin{array}{c} x^{2}\\ Ax^{2} \end{array}\right)\text{ with }\left(\begin{array}{c} x^{1}\\ Ax^{1} \end{array}\right),\left(\begin{array}{c} x^{2}\\ Ax^{2} \end{array}\right)\in \tilde{C}. \end{array}$$

Assuming *x* = *x*^1^ + *x*^2^ with *x*^1^, *x*^2^ ∈ *C* (and hence *Ax*^1^, *Ax*^2^, *Ax* ≥ 0), we have

$$\begin{array}{l} \text{ sign}\left(x^{1}\right),\text{sign}\left(x^{2}\right)\leq \text{sign}\left(x\right)\\ \;\Leftrightarrow \\ \text{sign}\left(\begin{array}{c} x^{1}\\ Ax^{1} \end{array}\right),\text{sign}\left(\begin{array}{c} x^{2}\\ Ax^{2} \end{array}\right)\leq \text{sign}\left(\begin{array}{c} x^{}\\ Ax^{} \end{array}\right). \end{array}$$

It remains to show the equivalence of the conclusions in the two definitions. In fact,

$$x^{1}=\lambda x^{2}\text{ with }\lambda >0\;\Leftrightarrow \;\left(\begin{array}{c} x^{1}\\ Ax^{1} \end{array}\right)=\lambda \left(\begin{array}{c} x^{2}\\ Ax^{2} \end{array}\right)\text{ with }\lambda >0.$$

                                                                                               □

Now, we can extend Theorem 3 to general polyhedral cones.

**Theorem 8**. *Let C* = {*x* ∣ *Ax* ≥ 0} *be a polyhedral cone. Every nonzero vector x ∈ C is a conformal sum of EVs. That is, there exists a finite set E ⊆ C of EVs such that*

$$x=\underset{e\in E}{\sum }e\;\mathrm{with}\text{ sign}\left(e\right)\leq \text{sign}\left(x\right).$$

*The set E can be chosen such that* |*E*| ≤ dim(*C*) *and* |*E*| ≤ |supp(*x*)| + |supp(*Ax*)|.

*Proof*. Let *A* ∈ ℝ^*m*×*r*^. Define the subspace

$$\tilde{S}=\left\{\left(\begin{array}{c} x^{}\\ Ax^{} \end{array}\right)\in {\mathbb{R}}^{r+m}|x\in \text{span}\left(C\right)\right\}$$

and the s-cone

$$\tilde{C}=\left\{\left(\begin{array}{c} x^{}\\ Ax^{} \end{array}\right)\in {\mathbb{R}}^{r+m}|x\in C\right\}.$$

Let *x* ∈ *C* be nonzero. By Theorem 3, $\left(\begin{array}{c} x^{}\\ Ax^{} \end{array}\right)\in \tilde{C}$ is a conformal sum of EVs. That is, there exists a finite set $Ẽ\subseteq \tilde{C}$ of EVs such that

$$\left(\begin{array}{c} x^{}\\ Ax^{} \end{array}\right)=\underset{\left(\begin{array}{c} e^{}\\ Ae^{} \end{array}\right)\in Ẽ}{\sum }\left(\begin{array}{c} e^{}\\ Ae^{} \end{array}\right)\text{ with sign}\left(\begin{array}{c} e^{}\\ Ae^{} \end{array}\right)\leq \text{sign}\left(\begin{array}{c} x^{}\\ Ax^{} \end{array}\right).$$

By Lemma 7, the EVs of *C* and $\tilde{C}$ are in one-to-one correspondence. Hence, there exists a finite set $E=\{e|\left(\begin{array}{c} e\\ Ae \end{array}\right)\in \;Ẽ\}$ ⊆ *C* of EVs such that

$$x=\underset{e\in E}{\sum }e\text{ with sign}\left(e\right)\leq \text{sign}\left(x\right).$$

The set Ẽ (and hence *E*) can be chosen such that $|E|=|Ẽ|\leq \text{dim}\left(\tilde{S}\right)=\text{dim}\left(C\right)$ and $\left|E|=|\tilde{E}|\leq |\text{supp}\left(\begin{array}{c} x\\ Ax \end{array}\right)|=|\text{supp}\left(x\right)|+|\text{supp}(Ax)\right|$.      □

Theorem 8 is a conformal refinement of Minkowski's and Carathéodory's theorems for polyhedral cones. In fact, it remains to show that there are finitely many EVs.

**Proposition 9**. *For a polyhedral cone, there are finitely many cND vectors up to positive scalar multiples*.

*Proof*. Let *C* be a polyhedral cone and $\tilde{C}$ the related s-cone. By Lemma 7, the cND vectors of *C* and $\tilde{C}$ are in one-to-one correspondence. By Proposition 5, the cND and SM vectors of $\tilde{C}$ coincide, and by Proposition 4, there are finitely many SM vectors.                         □

In Urbanczik and Wagner (2005), EVs of a polyhedral cone *C* were equivalently defined as extreme vectors of intersections of *C* with closed orthants of maximal dimension. Indeed, the following equivalence holds for closed orthants, not necessarily of maximal dimension.

**Proposition 10**. *Let C ⊆ ℝ^r^ be a polyhedral cone, x ∈ C, and O ⊂ ℝ^r^ a closed orthant with x ∈ O. Then*,

x∈C is cND ⇔ x∈C∩O is EX.

*Proof*. We show the equivalence of the premises in the definitions of conformal non-decomposability for *C* and extremity for *C* ∩ *O*. (The conclusions are identical.) Indeed, assuming *x* = *x*^1^ + *x*^2^, we have

$$\begin{array}{l} x^{1},x^{2}\in C\text{ with sign}\left(x^{1}\right),\text{sign}\left(x^{2}\right)\leq \text{sign}\left(x\right)\\ \;\Leftrightarrow \\ \;x^{1},x^{2}\in C\cap O. \end{array}$$

                                                                               □

### 3.3. Polyhedra

Let *P* be a polyhedron, that is,

$$P=\left\{x\in {\mathbb{R}}^{r}|Ax\geq b\right\}\text{ for some }A\in {\mathbb{R}}^{m\times r}\text{ and }b\in {\mathbb{R}}^{m}.$$

In order to extend Theorem 3 to polyhedra, we introduce corresponding special vectors.

#### 3.3.1. Special vectors

Let *P* be a polyhedron. A vector *x* ∈ *P* is called

- a *vertex*, if
  (VE) $$\begin{array}{lll} & \text{for all }x^{1},x^{2}\in P\text{ and }0<\lambda <1,\; & \;\\ & x=\lambda x^{1}+\left(1-\lambda \right)x^{2}\text{ implies }x^{1}=x^{2}, \end{array}$$
- and *convex-conformally non-decomposable*, if
  (ccND) $$\begin{array}{lll} & \text{for all }x^{1},x^{2}\in P\text{ with sign}\left(x^{1}\right),\text{sign}\left(x^{2}\right)\leq \text{sign}\left(x\right)\text{ and } & \;\\ & 0<\lambda <1,x=\lambda x^{1}+\left(1-\lambda \right)x^{2}\text{ implies }x^{1}=x^{2}. \end{array}$$

From the definitions, we have

VE⇒ccND.

For a polyhedral cone, we defined elementary vectors (EVs) via conformal non-decomposability. For a polyhedron, we require two sorts of EVs: convex-conformally non-decomposable vectors of the polyhedron and conformally non-decomposable vectors of its recession cone.

**Definition 11**. *Let P* = {*x* ∈ ℝ^*r*^ ∣ *Ax* ≥ *b*} *be a polyhedron and C^r^ = {x ∈ ℝ^r^ ∣ Ax* ≥ 0} *its recession cone. A vector e ∈ C^r^ ∪ P is called an* elementary vector of *P if either e ∈ C^r^ is conformally non-decomposable or e ∈ P is convex-conformally non-decomposable*.

In order to apply Theorem 3, we define an s-cone related to a polyhedron *P* = {*x* ∈ ℝ^*r*^ ∣ *Ax* ≥ *b*}. We introduce the *homogenization*

$$C^{h}=\left\{\left(\begin{array}{c} x\\ \xi \end{array}\right)\in {\mathbb{R}}^{r+1}|\xi \geq 0\text{ and }Ax-\xi b\geq 0\right\}$$

of the polyhedron, the subspace

$$\tilde{S}=\left\{\left(\begin{array}{c} x^{}\\ \xi^{}\\ Ax^{}-\xi^{}b \end{array}\right)\in {\mathbb{R}}^{r+1+m}|\left(\begin{array}{c} x\\ \xi \end{array}\right)\in \text{span}\left(C^{h}\right)\right\}$$

with $\text{dim}\left(\tilde{S}\right)=\text{dim}\left(C^{h}\right)=\text{dim}\left(P\right)+1$, and the s-cone

$$\begin{array}{l} \tilde{C}=C(\tilde{S},1+m)\\ \;=\{\left(\begin{array}{c} \overset{x}{\xi }\\ Ax-\xi b \end{array}\right)\in {\mathbb{R}}^{r+1+m}|\left(\begin{array}{c} x\\ \xi \end{array}\right)\in \text{span}(C^{h}),\;\xi \geq 0,\text{ and}\\ \;Ax-\xi b\geq 0\\ \;=\{\left(\begin{array}{c} \overset{x}{\xi }\\ Ax-\xi b \end{array}\right)\in {\mathbb{R}}^{r+1+m}|\left(\begin{array}{c} x\\ \xi \end{array}\right)\in C^{h}\} \end{array}$$

Hence,

$$\left(\begin{array}{c} x\\ \xi \end{array}\right)\in C^{h}\;\Leftrightarrow \;\left(\begin{array}{c} \overset{x}{\xi }\\ Ax-\xi b \end{array}\right)\in \tilde{C}.$$

Moreover, the cND vectors of *C^r^* and the ccND vectors of *P* (as the cND vectors of *C^h^*) are in one-to-one correspondence with the cND vectors of $\tilde{C}$.

**Lemma 12**. *Let P* = {*x* ∣ *Ax* ≥ *b*} *be a polyhedron, C^r^ = {x ∣ Ax ≥ 0} its recession cone, and*

$$\tilde{C}=\left\{\left(\begin{array}{c} x^{}\\ \xi^{}\\ Ax^{}-\xi^{}b \end{array}\right)\in {\mathbb{R}}^{r+1+m}|\xi \geq 0\text{ and }Ax-\xi b\geq 0\right\}$$

*the related s-cone. Then*,

$$x\in C^{r}\text{ is cND }\Leftrightarrow \;\left(\begin{array}{c} \underset{0}{x}\\ Ax \end{array}\right)\in \tilde{C}\text{ is cND}$$

and

$$x\in P\text{ is ccND }\Leftrightarrow \;\left(\begin{array}{c} \underset{1}{x}\\ Ax-b \end{array}\right)\in \tilde{C}\text{ is cND}.$$

*Proof*. See Appendix.                                     □

Now, we can extend Theorem 3 to polyhedra.

**Theorem 13**. *Let P* = {*x* ∣ *Ax* ≥ *b*} *be a polyhedron and C^r^* = {*x* ∣ *Ax* ≥ 0} *its recession cone. Every vector x ∈ P is a conformal sum of EVs. That is, there exist finite sets $E_{0}\subseteq C^{r}$ and *E*_1_ ⊆ *P* of EVs such that*

$$x=\underset{e\in E_{0}}{\sum }e+\underset{e\in E_{1}}{\sum }\lambda_{e}e\;\mathrm{with}\text{ sign}\left(e\right)\leq \text{sign}\left(x\right),$$

λ_e_ ≥ 0, and $\underset{e\in E_{1}}{\sum }\lambda_{e}=1$. (Hence, |*E*_1_| ≥ 1.)

*The set E = E_0_ ∪ E_1_ can be chosen such that* |*E*| ≤ dim(*P*) + 1 *and* |*E*| ≤ |supp(*x*)| + |supp(*Ax*)| + 1.

*Proof*. By defining an s-cone related to *P*, applying Theorem 3, and using Lemma 12. See Appendix.                                □

Theorem 13 is a conformal refinement of Minkowski's and Carathéodory's theorems for polyhedra. In fact, it remains to show that there are finitely many EVs.

**Proposition 14**. *For a polyhedron, there are finitely many ccND vectors*.

*Proof*. Let *P* be a polyhedron and $\tilde{C}$ the related s-cone. By Lemma 12, the ccND vectors of *P* are in one-to-one correspondence with a subset of cND vectors of $\tilde{C}$. By Proposition 5, the cND and SM vectors of $\tilde{C}$ coincide, and by Proposition 4, there are finitely many SM vectors.                 □

EVs of a polyhedron *P* can be equivalently defined as vertices of intersections of *P* with closed orthants.

**Proposition 15**. *Let P ⊆ ℝ^r^ be a polyhedron, x ∈ P, and O ⊂ ℝ^r^ a closed orthant with x ∈ O. Then*,

x∈P is ccND ⇔ x∈P∩O is VE.

*Proof*. We show the equivalence of the premises in the definitions of convex-conformal non-decomposability for *P* and of a vertex for *P* ∩ *O*. (The conclusions are identical.) Indeed, assuming *x* = λ*x*^1^ + (1 − λ)*x*^2^ with 0 < λ < 1, we have

$$\begin{array}{l} x^{1},x^{2}\in P\text{ with sign}(x^{1}),\text{sign}(x^{2})\leq \text{sign}(x)\\ \;\Leftrightarrow \\ \;x^{1},x^{2}\in P\cap O. \end{array}$$

                                                                              □

We conclude by noting that Theorem 8 is a special case of Theorem 13. If a polyhedron is also a cone, then *P* = *C*^*r*^, *E*_1_ = {0}, and $\underset{e\in E_{1}}{\sum }\lambda_{e}e=0$. However, we do not use Theorem 8 to prove Theorem 13. In classical proofs of Minkowski's and Carathéodory's theorems, one first studies polyhedral cones and then extends the results to polyhedra by a method called homogenization/dehomogenization; (see e.g., Ziegler, 1995).

### 3.4. Minimal generating sets

For a pointed polyhedral cone, the extreme rays form a minimal set of generators with respect to addition. The set is minimal in the sense that no proper subset forms a generating set and minimal in the even stronger sense that it is contained in every other generating set. Hence, the extreme rays form a *unique* minimal set of generators.

For a general polyhedral cone, there are minimal sets of generators (minimal in the sense that no proper subset forms a generating set), but there is no unique minimal generating set. However, there is a unique minimal set of *conformal* generators, namely the set of elementary vectors.

Recall that elementary vectors of a polyhedral cone are defined as conformally non-decomposable vectors. Indeed, every nonzero element of a polyhedral cone is a conformal sum of elementary vectors (Theorem 8), and every elementary vector is contained in a set of conformal generators.

We make the above argument more formal.

**Definition 16**. *Let C be a polyhedral cone. A subset G ⊆ C is called a* conformal generating set *if (i) every nonzero vector x ∈ C is a conformal sum of vectors in G, that is, if there exists a finite set G_x_ ⊂ G such that*

$$x=\underset{g\in G_{x}}{\sum }g\;\mathrm{with}\text{ sign}\left(g\right)\leq \text{sign}\left(x\right),$$

*and (ii) if λ G = G for all* λ > 0.

**Proposition 17**. *Let C be a polyhedral cone, E ⊆ C the set of elementary vectors, and G ⊆ C a conformal generating set. Then, E ⊆ G*.

*Proof*. Let *e* ∈ *C* be an elementary vector. Since *G* is a conformal generating set, we have

$$e=g^{*}+h\text{ with sign}\left(g^{*}\right),\text{sign}\left(h\right)\leq \text{sign}\left(x\right),$$

where we choose a nonzero $g^{*}\in G_{e}\subset G$ and write $h=\underset{g\in G_{e}\backslash \left\{g^{*}\right\}}{\sum }g\in C$. If |*G*_*e*_| = 1, then *h* = 0 and *e* = g^*^ ∈ *G*. Otherwise, since *e* is an elementary vector (a cND vector), we have *h* = λg^*^ with λ > 0 and hence *e* = (1 + λ)g^*^ ∈ *G*.                                    □

Analogously, for a polyhedron, there is a unique minimal set of conformal generators, namely the set of elementary vectors.

### 3.5. Examples

We illustrate our results by examples of polyhedral cones and polyhedra in two dimensions, and we return to the running example from the introduction.

**Example 1**. *The s-cone C* = {*x* ∣ *x*_1_ ≥ 0, *x*_2_ ≥ 0}.

**Figure:**
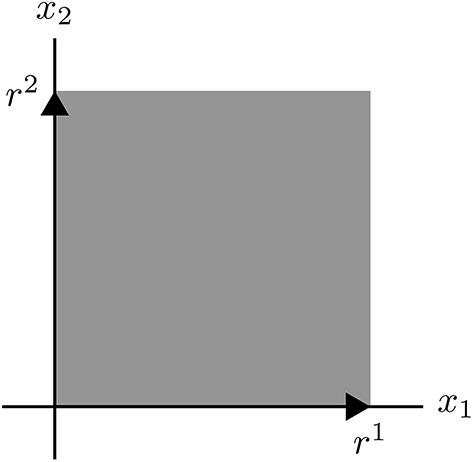


*Its EVs (SM vectors) are elements of the rays* $r^{1}=\left\{x|x_{1}>0,x_{2}=0\right\}$ *and* $r^{2}=\left\{x|x_{1}=0,x_{2}>0\right\}$ *(indicated by arrows). Every nonzero vector x ∈ C is a conformal sum of EVs. That is*,

$$x=e^{1}+e^{2},$$

*where e^1^ ∈ r^1^ and e^2^ ∈ r^2^*.

**Example 2**. *The general polyhedral cone*

$$C=\{x|\left(\begin{array}{cc} 3 & 1\\ -1 & 1 \end{array}\right)\left(\begin{array}{c} x_{1}\\ x_{2} \end{array}\right)\geq 0\}.$$

**Figure:**
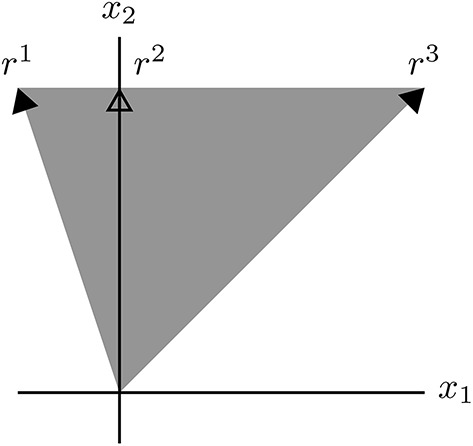


*Its EVs (cND vectors) are elements of the rays r*^1^, *r*^2^*, and r*^3^*. Note that r*^2^ *is not an extreme ray of C, but an extreme ray of* $C\cap {\mathbb{R}}_{\geq }^{2}$*, the intersection of the cone with the nonnegative orthant. Every nonzero vector x* ∈ *C is a conformal sum of EVs. In particular, if* $x\in C\cap {\mathbb{R}}_{\geq }^{2}$*, then*

$$x=e^{2}+e^{3},$$

*where e^2^ ∈ r^2^ and e^3^ ∈ r^3^*.

**Example 3**. *The polyhedron*

$$P=\{x|\left(\begin{array}{cc} 3 & 1\\ -3 & 3\\ 0 & 2 \end{array}\right)\left(\begin{array}{c} x_{1}\\ x_{2} \end{array}\right)\geq \left(\begin{array}{c} 1\\ -1\\ 1 \end{array}\right)\}.$$

**Figure:**
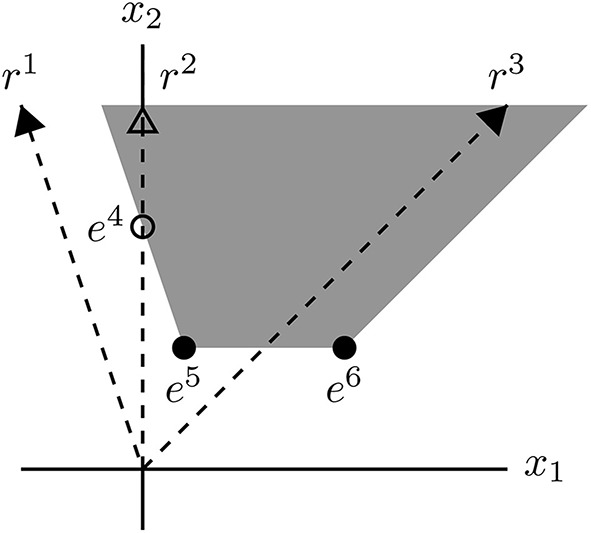


*Its EVs are elements of the rays r*^1^, *r*^2^, *and r*^3^ *(cND vectors of the recession cone) and the vectors e*^4^, *e*^5^, *and e*^6^ *(ccND vectors of the polyhedron). Note that e*^4^ *is not a vertex of P, but a vertex of $P\cap {\mathbb{R}}_{\geq }^{2}$, the intersection of the polyhedron with the nonnegative orthant. Every vector x ∈ P is a conformal sum of EVs. In particular, if $x\in P\cap {\mathbb{R}}_{\geq }^{2}$, then*

$$x=\left(e^{2}+e^{3}\right)+\left(\lambda_{4}e^{4}+\lambda_{5}e^{5}+\lambda_{6}e^{6}\right),$$

*where e*^2^ ∈ *r*^2^, *e*^3^ ∈ *r*^3^ *and* λ_4_, λ_5_, λ_6_ ≥ 0 *with* λ_4_ + λ_5_ + λ_6_ = 1.

Finally, we return to the running example from the introduction. We restate the underlying network, the corresponding stoichiometric matrix and the resulting flux cone:

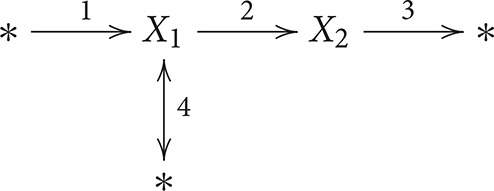



$$N=\left(\begin{array}{cccc} 1 & -1 & 0 & -1\\ 0 & 1 & -1 & 0 \end{array}\right),\;$$

$$\begin{array}{l} C=\left\{f\in {\mathbb{R}}^{4}|Nf=0\text{ and }f_{1},f_{2},f_{3}\geq 0\right\}. \end{array}$$

Its EVs (SM vectors) are

$$e^{1}=\left(\begin{array}{c} 1\\ 0\\ 0\\ 1 \end{array}\right),\;e^{2}=\left(\begin{array}{c} 0\\ 1\\ 1\\ -1 \end{array}\right),\;e^{3}=\left(\begin{array}{c} 1\\ 1\\ 1\\ 0 \end{array}\right),$$

and their positive multiples. In other words, the EVs are elements of the rays *r*^1^ = {λ*e*^1^ ∣ λ > 0}, *r*^2^ = {λ*e*^2^ ∣ λ > 0}, and *r*^3^ = {λ*e*^3^ ∣ λ > 0}.

The flux cone is defined by the stoichiometric matrix and the set of irreversible reactions. If additionally lower/upper bounds for the fluxes through certain reactions are known, then one is interested in the resulting flux polyhedron. In the example, we add an upper bound for the flux through reaction 1, in particular, we require *f*_1_ ≤ 2 and obtain the flux polyhedron

$$P=\left\{f\in {\mathbb{R}}^{4}|Nf=0,f_{1},f_{2},f_{3}\geq 0,\text{ and }f_{1}\leq 2\right\}.$$

Its EVs are elements of the ray *r*^2^ = {λ*e*^2^ ∣ λ > 0} (cND vectors of the recession cone) and the vectors *e*^1^, *e*^3^, *e*^4^ (ccND vectors of the polyhedron), where

$$e^{1}=\left(\begin{array}{c} 2\\ 0\\ 0\\ 2 \end{array}\right),\;e^{2}=\left(\begin{array}{c} 0\\ 1\\ 1\\ -1 \end{array}\right),\;e^{3}=\left(\begin{array}{c} 2\\ 2\\ 2\\ 0 \end{array}\right),\;e^{4}=\left(\begin{array}{c} 0\\ 0\\ 0\\ 0 \end{array}\right).$$

Note that *e*^3^ is not a vertex of *P*, but a vertex of $P\cap {\mathbb{R}}_{\geq }^{4}$, the intersection of the polyhedron with the nonnegative orthant. Every vector *x* ∈ *P* is a conformal sum of EVs. In particular, if $x\in P\cap {\mathbb{R}}_{\geq }^{4}$, then

$$x=\lambda_{1}e^{1}+\lambda_{3}e^{3}+\lambda_{4}e^{4},$$

where λ_1_, λ_3_, λ_4_ ≥ 0 with λ_1_ + λ_3_ + λ_4_ = 1. In other words, the polyhedron $P\cap {\mathbb{R}}_{\geq }^{4}$ is a polytope.

In applications such as computational strain design, the set of EVs (the unique minimal set of *conformal* generators) is often more useful than a minimal set of generators. In the example, the set of EVs includes *e*^3^ which is a ccND vector, but not a vertex of *P*. If we delete reaction 4 by gene knockout, the new set of EVs consists of *e*^3^ and *e*^4^ (having zero flux through reaction 4), and the resulting flux polyhedron is the polytope generated by *e*^3^ and *e*^4^. Most importantly, we obtain the result without recalculating the set of generators (after deleting reaction 4).

## 4. Discussion

Metabolic pathway analysis aims to identify meaningful routes in a network, in particular, to decompose fluxes into *minimal* metabolic pathways. However, only a decomposition *without cancelations* is biochemically meaningful, since a reversible reaction cannot have a flux in different directions at the same time.

In mathematical terms, one is interested in a *conformal* decomposition of the flux cone and of general polyhedral cones and polyhedra. In this work, we first study s-cones (like the flux cone) arising from a linear subspace and nonnegativity conditions. Then, we analyze general polyhedral cones and polyhedra via corresponding higher-dimensional s-cones. Without assuming previous knowledge of polyhedral geometry, we provide an elementary proof of a conformal refinement of Minkowski's and Carathéodory's theorems (Theorems 3, 8, and 13): Every vector (of an s-cone, a general polyhedral cone, or a polyhedron) is a conformal sum of *elementary vectors* (conformally non-decomposable vectors), and there is an upper bound on the number of elementary vectors needed in a conformal decomposition (in terms of the dimension of the cone or polyhedron).

As a natural next question, one may ask: what is a *minimal generating set* of a polyhedral cone that allows a conformal decomposition of every vector? Clearly, such a set must contain all conformally non-decomposable vectors. Indeed, we show that the elementary vectors form a *unique* minimal set of conformal generators (Proposition 17). In metabolic pathway analysis, the question is: what is a minimal generating set of the flux cone that allows a biochemically meaningful decomposition of every flux mode? In this case, the *elementary modes* form a unique minimal set of generators without cancelations. This property distinguishes elementary modes as a fundamental concept in metabolic pathway analysis and may serve as a definition.

The correspondence of general polyhedral cones and polyhedra to higher-dimensional s-cones has also important consequences for the *computation* of elementary vectors. In particular, it allows to use efficient algorithms and software developed for elementary modes (see e.g., Zanghellini et al., 2013 and the references therein) for computing elementary vectors of general polyhedral cones and polyhedra.

In *applications*, decompositions without cancelations were first used in the study of the conversion cone Urbanczik and Wagner (2005), a general polyhedral cone obtained by flux cone projection Marashi et al. (2012). The approach was extended to polyhedra arising from the flux cone and inhomogeneous constraints, in particular, to describe the solution set of linear optimization problems encountered in flux balance analysis Urbanczik (2007). In analogy to s-cones, these sets could be called s-polyhedra. Recently, elementary vectors have been used to describe such polyhedra in the study of growth-coupled product synthesis Klamt and Mahadevan (2015). Interestingly, conformal decompositions of the flux cone itself appeared rather late. In fact, they have been used to characterize optimal solutions of enzyme allocation problems in *kinetic* metabolic networks Müller et al. (2014).

Minkowski's and Carathéodory's theorems (and their conformal refinements) are fundamental results in polyhedral geometry with important applications in metabolic pathway analysis. In subsequent work, we plan to revisit other results from polyhedral geometry and oriented matroids (like Farkas' lemma) and investigate their consequences for metabolic pathway analysis.

## Author contributions

The authors contributed equally to the work and approved it for publication.

### Conflict of interest statement

The authors declare that the research was conducted in the absence of any commercial or financial relationships that could be construed as a potential conflict of interest.

## Footnotes

^*^For a counterexample, consider the subspace *S* = ker(1, −1, −1, 1)⊆ℝ^4^. Its nonnegative EVs are

$$e^{1}=\left(\begin{array}{c} 1\\ 1\\ 0\\ 0 \end{array}\right),\;e^{2}=\left(\begin{array}{c} 1\\ 0\\ 1\\ 0 \end{array}\right),\;e^{3}=\left(\begin{array}{c} 0\\ 1\\ 0\\ 1 \end{array}\right),\;e^{4}=\left(\begin{array}{c} 0\\ 0\\ 1\\ 1 \end{array}\right),$$

and their positive multiples. Then *x* = (1, 2, 3, 4)^*T*^ is not a conformal sum of EVs with the claimed property. (Every conformal decomposition of *x* consists of at least 3 EVs, and every set of 3 EVs contains 1 EV which does not have a nonzero component which is zero in the other EVs.)

## Appendix

We prove the main results for polyhedra, Lemma 12 and Theorem 13.

*Proof of Lemma 12*. To prove the first equivalence, we note that $\left(\begin{array}{c} x^{}\\ 0\\ Ax^{} \end{array}\right)\in \tilde{C}$ is cND if and only if $\left(\begin{array}{c} x\\ Ax \end{array}\right)$ ∈ *C*′ is cND, where *C*′ = {$\left(\begin{array}{c} x\\ Ax \end{array}\right)$ ∈ ℝ^*r*+*m*^ ∣ *Ax* ≥ 0}, and apply Lemma 7.

To prove the second equivalence, we show the two implications separately:

(⇒) We assume that *x* ∈ *P* is ccND and first consider a conformal sum of the form

$$\left(\begin{array}{c} x^{}\\ 1\\ Ax^{}-b \end{array}\right)=\left(\begin{array}{c} x^{1}\\ 1\\ Ax^{1}-b \end{array}\right)+\left(\begin{array}{c} x^{2}\\ 0\\ Ax^{2} \end{array}\right)$$

with *x*^1^ ∈ *P*, nonzero *x*^2^ ∈ *C*^*r*^, and sign(*x*^1^), sign(*x*^2^) ≤ sign(*x*). Indeed, we also have $x=\frac{1}{2}x^{1}+\frac{1}{2}\left(x^{1}+2x^{2}\right)$ with *x*^1^, *x*^1^ + 2*x*^2^ ∈ *P* and sign(*x*^1^), sign(*x*^1^ + 2*x*^2^) ≤ sign(*x*). By the assumption, *x*^1^ = *x*^1^ + 2*x*^2^, that is, *x*^2^ = 0, and it remains to consider a conformal sum of the form

(+) $$\left(\begin{array}{c} x^{}\\ 1\\ Ax^{}-b \end{array}\right)=\lambda \left(\begin{array}{c} x^{1}\\ 1\\ Ax^{1}-b \end{array}\right)+\left(1-\lambda \right)\left(\begin{array}{c} x^{2}\\ 1\\ Ax^{2}-b \end{array}\right)$$

with *x*^1^, *x*^2^ ∈ *P*, sign(*x*^1^), sign(*x*^2^) ≤ sign(*x*), and 0 < λ < 1. By the assumption, *x*^1^ = *x*^2^, and the first vector in the sum is a positive multiple of the second. That is,

(*) $$\lambda \left(\begin{array}{c} x^{1}\\ 1\\ Ax^{1}-b \end{array}\right)=\mu \left(1-\lambda \right)\left(\begin{array}{c} x^{2}\\ 1\\ Ax^{2}-b \end{array}\right)$$

with μ > 0. Hence, $\left(\begin{array}{c} x^{}\\ 1\\ Ax^{}-b \end{array}\right)\in \tilde{C}$ is cND.

(⇐) We assume that $\left(\begin{array}{c} x^{}\\ 1\\ Ax^{}-b \end{array}\right)\in \tilde{C}$ is cND and consider the convex-conformal sum

$$x=\lambda x^{1}+\left(1-\lambda \right)x^{2}$$

with *x*^1^, *x*^2^ ∈ *P*, sign(*x*^1^), sign(*x*^2^) ≤ sign(*x*), and 0 < λ < 1. Hence, we also have the conformal sum (+). By the assumption, we have equation (*) which implies *x*^1^ = *x*^2^. Hence, *x* ∈ *P* is ccND.                       □

*Proof of Theorem 13*. Let *A* ∈ ℝ^*m*×*r*^ and *b* ∈ ℝ^*m*^. Define the homogenization

$$C^{h}=\left\{\left(\begin{array}{c} x\\ \xi \end{array}\right)\in {\mathbb{R}}^{r+1}|\xi \geq 0\text{ and }Ax-\xi b\geq 0\right\},$$

the subspace

$$\tilde{S}=\left\{\left(\begin{array}{c} x^{}\\ \xi^{}\\ Ax^{}-\xi^{}b \end{array}\right)\in {\mathbb{R}}^{r+1+m}|\left(\begin{array}{c} x\\ \xi \end{array}\right)\in \text{span}\left(C^{h}\right)\right\}$$

and the s-cone

$$\tilde{C}=\left\{\left(\begin{array}{c} x^{}\\ \xi^{}\\ Ax^{}-\xi^{}b \end{array}\right)\in {\mathbb{R}}^{r+1+m}|\left(\begin{array}{c} x\\ \xi \end{array}\right)\in C^{h}\right\}.$$

Let *x* ∈ *P*. By Theorem 3, $\left(\begin{array}{c} x^{}\\ 1\\ Ax^{}-b \end{array}\right)\in \tilde{C}$ is a conformal sum of EVs. That is, there exist finite sets ${Ẽ}_{0},{Ẽ}_{1}\subseteq \tilde{C}$ of (normalized) EVs such that

$$\left(\begin{array}{c} x^{}\\ 1\\ Ax^{}-b \end{array}\right)=\underset{\left(\begin{array}{c} e_{}\\ 0\\ Ae_{} \end{array}\right)\in {Ẽ}_{0}}{\sum }\left(\begin{array}{c} e_{}\\ 0\\ Ae_{} \end{array}\right)+\underset{\left(\begin{array}{c} e_{}\\ 1\\ Ae_{}-b \end{array}\right)\in {Ẽ}_{1}}{\sum }\lambda_{e}\left(\begin{array}{c} e_{}\\ 1\\ Ae_{}-b \end{array}\right)$$

with

$$\text{sign}\left(\begin{array}{c} e_{}\\ 0\\ Ae_{} \end{array}\right),\text{sign}\left(\begin{array}{c} e_{}\\ 1\\ Ae_{}-b \end{array}\right)\leq \text{sign}\left(\begin{array}{c} x^{}\\ 1\\ Ax^{}-b \end{array}\right),$$

λ_*e*_ ≥ 0, and $\underset{e\in E_{1}}{\sum }\lambda_{e}=1$. By Lemma 12, the EVs of *P* are in one-to-one correspondence with the EVs of $\tilde{C}$. Hence, there exist finite sets $E_{0}=\left\{e|\left(\begin{array}{c} e_{}\\ 0\\ Ae_{} \end{array}\right)\in {Ẽ}_{0}\right\}\subseteq C^{r}$ and *E*_1_ = {*e* ∣ $\left(\begin{array}{c} e\\ 1\\ Ae-b \end{array}\right)$ ∈ Ẽ_1_} ⊆ *P* of EVs such that

$$x=\underset{e\in E_{0}}{\sum }e+\underset{e\in E_{1}}{\sum }\lambda_{e}e\text{ with sign}\left(e\right)\leq \text{sign}\left(x\right).$$

The set Ẽ = Ẽ_0_ ∪ Ẽ_1_ (and hence *E* = *E*_0_ ∪ *E*_1_) can be chosen such that $|E|=|Ẽ|\leq \text{dim}\left(\tilde{S}\right)=\text{dim}\left(P\right)+1$ and |*E*| = |Ẽ| ≤ |supp $\left(\begin{array}{c} x\\ 1\\ Ax-b \end{array}\right)$| = |supp(*x*)| + 1 +|supp(*Ax* − *b*)|.                                        □
